# Cattle’s Social Rank Throughout the Transition from Rangeland to Fattening Affects Beef Quality

**DOI:** 10.3390/ani15121690

**Published:** 2025-06-07

**Authors:** Paola Soberanes-Oblea, Iván Adrián García-Galicia, Mariana Huerta-Jiménez, Jesús Ricardo Gámez-Piñón, Mieke Titulaer, Alma Delia Alarcon-Rojo, Einar Vargas-Bello-Pérez

**Affiliations:** 1 Facultad de Zootecnia y Ecología, Universidad Autónoma de Chihuahua, Chihuahua 31000, Mexico; p366588@uach.mx (P.S.-O.); mhuertaj@uach.mx (M.H.-J.); jgamez@uach.mx (J.R.G.-P.); mieke.t@martineztorre.tecnm.mx (M.T.); 2 C.E.I.E.G.T., Facultad de Medicina Veterinaria y Zootecnia, Universidad Nacional Autónoma de México, Tlapacoyan 93650, Mexico; igarciag@unam.mx; 3 Instituto Tecnológico Superior de Martinez de la Torre, Tecnológico Nacional de México, Martinez de la Torre 93610, Mexico

**Keywords:** beef quality, ranking hierarchy, pre-slaughter stress, cattle behavior, beef color

## Abstract

The social stress due to regrouping and hierarchy establishment in cattle in the transition from rangeland to feedlots may cause considerable losses in beef production due to aggressive interactions. This study determined the effect of the premortem social ranks of bovines in the transition from rangelands to fattening pens on beef quality. The behavior of 20 heifers was evaluated in three premortem stages: 72 h after transportation, after 6 weeks of fattening, and while waiting at the slaughterhouse. Heifers were classified as dominant (D) or subordinate (S) based on ethograms. The principal component analysis revealed a strong relationship between social rank and beef color (L*, a*, b*, chroma, HUE, and ΔE), pH, and water holding capacity. The linear discriminant analysis of the beef quality variables classified the animals in one of four social ranks (dominant, change to subordinate, subordinate, change to dominant) with 95% precision. pH 45 (−9.163), drip 0 d (−3.917), final chroma (1.773), and ΔE (1.646) obtained high coefficients that determined the separation among the classes. Color variables like ΔE (*p* = 0.049) and chroma 0 d (*p* = 0.053) showed significant differences between the D and S animals in the MANOVA. Reactivity in dominants tended to negatively impact beef quality, affecting its color and pH. These findings contribute to a better understanding of how social dynamics impact beef quality during the transition period.

## Simple Summary

Regrouping cattle during pre-slaughter handling disrupts their social order, leading to social stress, especially when cattle move from rangeland to feedlots, affecting beef production. We observed the behavior of 20 heifers at three key points: 72 h after transportation, six weeks during fattening, and while they waited at the slaughterhouse. The heifers were classified as dominant (D) or subordinate (S) based on their behaviors. Key factors used to distinguish between groups included pH at 45 min postmortem, drip loss at 0 days, final chroma, and change in color. A strong link between social rank and various aspects of beef quality such as color, pH, and water holding capacity, was found. The discoloration and chroma of beef were different between the dominant and subordinate animals. The dominant heifers tended to show behavior that negatively influenced beef quality, such as its color and pH. Social rank plays a large role in how cattle behave and beef is produced.

## 1. Introduction

Transitioning cattle to a feedlot is a critical process because they experience significant changes in their environment, diet, and management. Adaptation is considered the most critical period for feedlot cattle because it is a period of transition from a forage-based diet to a high-concentrate diet [1]. Regarding their management, livestock establish social interaction behaviors, described by dominant–subordinate relationships [2]. The establishment of this social rank is natural and depends highly on the age of the animals [3,4]. However, when ages are similar within beef feedlots, this establishment depends on agonistic behaviors [5] that can sometimes cause stress, bruising, and physical injuries [2,6]. On the other hand, grooming [7], proximity [8], and playing [9] are behaviors that help repair relationships after possibly stressful events [10]. Stable social ranks reduce the level of aggressiveness within a group, favor production [11], and benefit the quality of the meat from these animals [12].

Social stress during the pre-slaughter period is a major concern for animal welfare associations, producers, and consumers. The presence of social aggression or fights among animals because of competition frequently leads to poor-quality beef [13]. This behavior is probably more common in animals raised in free conditions and are then fattened under indoor conditions. Visual defects such as bruises on the carcass and changes in biochemistry, such as an increase in cortisol or lactate, which leads to a high final pH, reductions in water holding capacity, and decreased tenderness in beef, have been described in meat from cattle that experienced social stress related to mixing or fighting before slaughter [6,14,15].

Studies of social dominance behavior are relevant to the management of cattle because they provide information on their susceptibility to social stress and its possible effects on productive traits such as beef quality [12]. Thus, the objective of this study was to characterize the social rank and behavior of cattle during the transition period and to determine their effect on productive performance. We hypothesized that pre-slaughter stress derived from social mixing and the social rank (dominant or subordinate) of the animal before slaughter would affect beef quality parameters. It is important to note that no other studies are available on cattle’s social rank and its effects on beef quality for animals raised under free conditions in mountain rangelands and then put into a feedlot system. This study was performed during different stages, and the animals’ behaviors were followed during these stages. These behaviors were then related to beef quality. Farmers raising animals under these conditions will benefit from the data from this study, as they can then make more precise management decisions during transportation, reception, and fattening.

## 2. Materials and Methods

### 2.1. The Animals and the Study Site

The animal study protocol was approved by the Postgraduate Department and by the Animal Ethics and Welfare Committee of the Faculty of Animal Science and Ecology of the Autonomous University of Chihuahua. This study was carried out using cattle from the Research and Technology Transfer Center located in the town of Teseachi, Chihuahua, Mexico (28°48′ N; 107°25′ E). The study site is a transition zone of forest–grassland with the presence of native vegetable species such as *Bouteloua gracilis*, *Lycurus phleoides*, *Muhlenbergia rígida*, *Bouteloua hirsuta*, *Elynorus barbiculmis*, and *Muhlenbergia rigida*. Twenty heifers each weighing approximately 400 kg (Angus × Hereford × Criollo Raramuri) were kept for more than 14 months on the rangeland with free access to water and native pastures in a rotational grazing system of paddocks of approximately 135 ha^2^. The animals were well adapted to grazing under mountain conditions in extensive areas with deep inclinations (with a 10–30% slope) for weeks (4–12 weeks) without contact with humans. Later, the animals were transported to the SASE, located in Chihuahua City. The behavioral observations were carried out at three different stages; the initial stage involved monitoring the animals the first 72 h after a 6 h transportation period from the rangeland to fattening pens. Observations were conducted for 20 min per pen (five animals per pen), over a total of four hours in the morning and four hours in the afternoon. The animals were housed randomly in groups of 5 animals divided over 4 reception pens (15 × 15 m) with ad libitum access to water and a balanced diet according to the NRC [16], provided in concrete feeders with dimensions of 15 l × 1.0 w × 0.60 h, m. The animals were kept in these conditions until the day of processing. Finally, the third stage evaluated the waiting time in the pen after their transport to the slaughterhouse, with an observation period of 1 h [17,18].

Behavioral observations were conducted by five trained observers based on an ethogram of relevant behaviors, including agonistic and non-agonistic interaction behaviors, as well as individual behaviors (Table 1A) [19,20,21].

Agonistic interactions were counted to define the Dominance Index (DI) for each animal in each of the three stages of the study according to Vargas-Bello-Pérez [22] and Galindo and Broom [23]. Encounters won (aggressive interactions initiated) and lost (aggressive interactions participated in without initiating) were considered to calculate the DI. Heifers can be categorized as subordinate or dominant based on their DI value, which is a measure of their position within a social hierarchy. A DI value below 0.40 typically indicates a subordinate (low-ranking) animal, while a value of 0.60 or higher suggests a dominant (high-ranking) animal [23] (Table 1B). All observations were noted in real time, as a video analysis was not used.

The categories “change to dominant” and “change to subordinate” in the observed cattle were established by monitoring social interactions that showed changes in the dominance hierarchy. This was carried out by recording interactions between individuals during the observation periods and analyzing changes in the group hierarchy. That is, if an individual that initially displayed subordinate behaviors (avoiding conflict or retreating from another animal) begins to display dominance behaviors (displacing another animal, fighting, or butting to gain resources such as space and feed) more frequently toward certain individuals, it can be classified as a “change to dominant”. If a dominant individual relinquishes its position and displays submissive behaviors toward individuals it previously subordinated, this is classified as a “change to subordinate”.

### 2.2. The Beef Quality Analysis

Beef quality characteristics were evaluated in the longissimus thoracis muscle from the heifers, which is a more studied muscle in meat evaluations. Visible fat was manually removed, and the samples were cut into 2.5 cm thick steaks in duplicate 24 h postmortem (2 °C). The steaks were vacuum-packed and aged at 4 °C for 15 d. They were subsequently stored in an ultra-freezer at 80 °C for the same duration of time. Physicochemical analyses were carried out on the day of thawing and after 5 d of simulated retail display (with 20 min of blooming).

The pH of the beef was recorded at 45 min and 24 h postmortem, as well as at day 0 of shelf-life, using a digital pH meter (CR-400 Hanna, Cluj-Napoca, Romania), previously calibrated according to the manufacturer’s instructions. The measurements were taken directly on the beef steaks in triplicate according to the method by Honikel [24].

The color was determined on days 0 and 7 using a colorimeter (Konica Minolta, CR 400, Tokyo, Japan; Illuminant C; 2° observer angle) according to the CIELab methodology [25]. The color of the steaks was evaluated directly in triplicate. Values were expressed as L* (luminosity), a* (+red to −green), b* (+yellow to −blue), C* (chroma), HUE (hue angle), and ∆E (difference in color).

The water holding capacity (WHC) was determined through the compression method by Tsai and Ockerman [26] on days 0 and 7 of shelf-life. A 0.3 g sample was weighed on an analytical balance and placed between two plexiglass squares (15 × 15 cm) so that a constant pressure of 10 kg could be applied for 20 min. The WHC was expressed as the percentage difference between the sample before and after compression. The drip loss was evaluated following the methodology of Honikel and Hamm [24]. Three grams (±0.5) of the sample was suspended inside a container and stored for 48 h at 4 °C. The drip loss was calculated by the weight difference.

Determination of the shear force was carried out according to the AMSA methodology [27] using a Warner Bratzler “V”-shaped blade (with a 60° triangular opening) and a TA-TX-plus texture analyzer (Stable Micro Systems Ltd., Surrey, UK) at a speed of 2.0 mm/s. The steak samples were cooked on electric grills (George Foreman^®^, Middleton, WI, USA) until they reached 70 ± 1 °C at their geometric centers. The temperature was monitored using a thermocouple inserted at the beginning of the cooking process, which was connected to an infrared thermometer (Fisherbrand^®^, Mod. 90012, Waltham, MA, USA). Subsequently, the samples were stored at 4 °C for 24 h. At least seven cylinders (with a diameter of 10.0 mm) from every steak were obtained parallel to the longitudinal orientation of the muscle fibers. The maximum force to shear was recorded transversely in each cylinder, and the average was reported in Newtons.

### 2.3. The Statistical Analysis

The data were subjected to a principal component analysis (PCA), a linear discriminant analysis, and a multivariate analysis of variance (MANOVA) using the statistical program R, version 4.4.2. [28]. The PCA and MANOVA were performed using the stats package part of R. The MASS package was used to perform the linear discriminant analysis [29]. The package Factoextra was used to visualize the results of the PCA [30], while ggpubr was used to create the box plots [31].

## 3. Results

**Figure 1:**
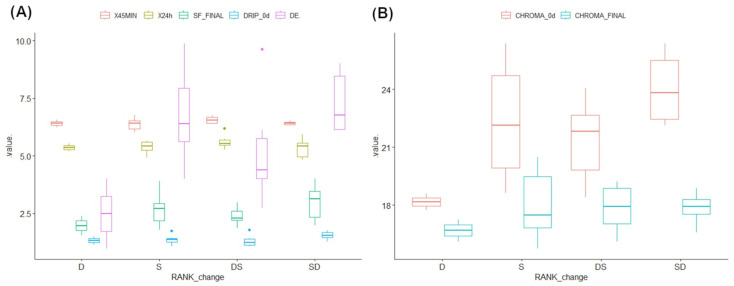
Box plots of beef quality variables from animals classified as dominant (D); dominant changing to subordinate (DS); subordinate (S); or subordinate changing to dominant (SD). (**A**) Values on the y axis: (**A**) X45MIN = pH 45 min postmortem; X24h = pH 24 h postmortem; SF = final shear force; DRIP_0d = drip loss 0 d postmortem; and DE = total change in color after display. (**B**) CHROMA_0d = CHROMA 0 d postmortem; CHROMA_FINAL = final chroma value.

The physicochemical values for the longissimus thoracis were within the normal limits (Table 2). Data from only one animal were high but not necessarily atypical. Throughout the three evaluation stages, the animals maintained or changed their social ranks. Only two animals remained dominant (D) during the three stages, which was associated with pH (45 min and 24 h). Meanwhile, animals changing from dominant to subordinate (DS) had a higher association with their WHC, pH, and color variables (Figure 1). Only two animals remained dominant (D) during the three stages and they were associated to pH (45 min and 24 h). Meanwhile, animals changing from dominant to subordinate (DS) had a higher association with their WHC, pH, and color variables (Figure 1).

### 3.1. The Principal Component Analysis

**Figure 2:**
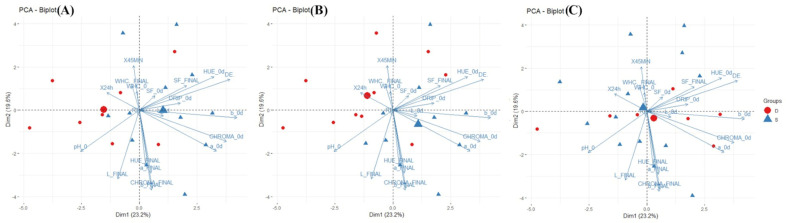
A biplot of the PCA with the physicochemical traits of the longissimus thoracis at (**A**) 72 h, (**B**) 6 weeks of fattening, and (**C**) slaughterhouse reception/waiting.

The PCA resulted in the extraction of six components with an eigenvalue of >1. PC 1 explained 23.2% of the variance and was negatively associated with pH 0 d and positively associated with a* final, b* final, b* 0 d, hue 0 d, chroma 0 d, and ∆E (with PCA loadings > 0.30). PC 2 explained 19.6% of the variance and was negatively associated with all of the final color characteristics (L*, a*, b*, hue, and chroma), that is, with the color of the meat measured after 5 days on the shelf. The results for PC 1 and 2 were plotted onto a biplot (Figure 2) to visualize the relationship between beef quality and social rank at the three different stages of the study (72 h: Figure 2A; 6 weeks: Figure 2B; pre-processing: Figure 2C). Figure 2 also shows the separation of the two ranks based on PC 1 and 2, evidencing that each social order is related to specific beef characteristics.

At the 72 h stage (Figure 2A), the dominant animals are associated with higher pH values (0, 24 h, and 45 min). At the 6-week stage (Figure 2B), subordinate animals seem to have an association with higher values for beef quality variables such as a* 0 d, b* 0 d, chroma 0 d, b* final, L* final, chroma final, ΔE, and hue final, while dominant animals show similar associations to those in the previous stage. Finally, in the pre-slaughter stage (Figure 2C), the distribution of dominant animals is associated with higher values for color variables such as a* 0 d, b* 0 d, and chroma 0 d. The color of beef after slaughter and after aging seems to be the main factor affected by the hierarchy behavior of the cattle during the fattening and before slaughter.

### 3.2. The Linear Discriminant Analysis (LDA)

To identify which beef quality variables were most relevant to differentiating social ranks, a linear discriminant analysis (LDA) was performed to classify the animals (*n* = 20) into the four different ranks (D, DS, S, and SD) using the beef quality variables as predictors (pH 45 min, pH 24 h, final shear force, WHC 0 d, dripping 0 d, chroma 0 d, final chroma, and ΔE; Figure 1). The model achieved an accuracy of 95%. Only one SD animal was incorrectly classified as DS. The variables with the highest coefficients for the first linear discriminant (LD1) were pH 45 (−9.16), drip 0 d (−3.92), final chroma (1.77), ∆E (1.65), and chroma 0 d (−1.09). The absolute value of a coefficient reflects the strength of the relationship in the discriminant function.

### 3.3. The Analysis of the Impact of Social Rank on Beef Quality Variables (MANOVA)

The MANOVA indicated an effect of social rank (D = dominant, S = subordinate, SD = change from subordinate to dominant, DS = change from dominant to subordinate) on beef quality variables such as pH 45, pH 24, final shear force, WHC 0 d, final WHC, drip 0 d, chroma 0 d, final chroma, and ΔE (*p* = 0.057).

Based on univariate tests, the ΔE values were lower (*p* = 0.049) in animals classified as D, followed by DS, S, and SD (Figure 1A). The loss of beef’s color was less frequently observed in animals that were dominant or that were dominant at the beginning of the evaluations.

## 4. Discussion

This study was performed during different stages, and the animals’ behaviors were followed up during these stages. Then, these behaviors were related to beef quality. It is important to note that overall, animals changing from subordinate to dominant (SD) were associated with beef color, influencing a*, b*, and chroma at 0 d. In this regard, the type of group housing may influence perceptions of beef’s color due to stress since it modifies the use of glucose before slaughter, which modifies the metabolism of the muscle fibers and pH, directly impacting the color through phenomena such as DFD (Dry, Firm, and Dark) or PSE (Pale, Soft, and Exudative) [14,32]. Furthermore, the animals used in this study were kept under grazing conditions and then moved to indoor facilities, resulting in a change in dominance, partly due to the energy invested in maintaining their hierarchical position in reduced spaces, as noted by another reviewer [33], thus affecting the color of their meat.

Immonen et al. [34] also reported a relationship between pH and stress, although not severe enough to cause obvious changes in color, such as dark cuts. Stress levels depend on numerous factors, with the period before slaughter being one of the most significant [34]. Andrés-Bello et al. [35] stated that there cannot be a generalized influence of pH on the remaining physicochemical characteristics. Herrán et al. [36] mentioned that transportation and waiting in pens increase aggressive behaviors. Excitability and flight in animals from low social ranks have an impact on the sensory attributes of beef [2].

The pH of beef is influenced by the degradation of glycogen in response to physical and psychological stress factors [37]. It can be observed that lower pH measurements are associated with a higher prediction probability. Drip loss is highly associated with short, intense stress before slaughter. Prolonged stress causes DFD beef characterized by a high pH and a strong water-binding capacity, resulting in meat that appears dark and dry, even after cooking. High loss of water during the post-slaughter period causes the lixiviation of myoglobin, the natural pigment in meat, leading to discoloration and an undesirable texture [14]. This was probed by Carrasco-García et al. [14], who compared two groups of bovine carcasses exposed to premortem stress with different pHs, highlighting the color values related to dark cuts as the main difference.

A change in beef’s color can be caused by an increase in physical and social activity [38] and the relationship with the storage time of the beef [39]. Chroma 0 d (*p* = 0.053) did not differ among hierarchy statuses, although lower values were observed in the dominant animals (Figure 1B). A decrease in chroma values during storage has been strongly related to poor handling, which leads to an increase in cortisol in the moments premortem [14,40]. Color deficiencies in DFD beef are directly associated with the stress experienced by the animals in postmortem handling and glycogen expenditure during this stressful handling [14]. Furthermore, elevated plasma cortisol levels are also associated with inadequate livestock handling [40]. Therefore, proper animal welfare during growth, mobilization, and slaughter prevents undesirable changes in beef’s color [41,42,43]. The increased activity and physical wear observed in dominant animals, especially when related to maintaining their position, are strong indicators of social stress and its connection to hierarchical position, and this depends on dominance activity and stability [17,44].

Assessing behavior alone can limit the information about the stress faced by each social rank. Although both groups (subordinate and dominant) presumably experienced stress, this may have manifested itself both physically and psychologically. Therefore, complementing these types of experiments with multimodal indicators, such as the use of biomarkers, could offer a more comprehensive view of the intensity of the stress experienced by the animals. However, the advantage of the present study is that the evaluations were carried out using non-invasive indicators, which did not cause additional stress for the animals and removed any bias related to induced stress. This allowed for repetition of the sampling and ensured the veracity of the results. Future studies could include the breed and sex of the animals to acquire a broader picture of the variables related to social stress and beef quality.

It is worth saying that this study used a relatively low number of animals; however, we analyzed and arranged a robust dataset that included behavior and meat quality data. Further studies should consider expanding the number of animals.

## 5. Conclusions

Social rank was related to beef quality parameters based on different analysis procedures (PCA, LDA, and MANOVA). Important beef quality variables were related to social ranking. Animals changing from dominant to subordinate had a higher association with the color parameters, pH, and WHC. Therefore, it can be concluded that social stress related to hierarchy during the fattening period or on arrival to slaughter is important to beef quality in livestock, particularly in terms of pH and color. This study shows the importance of social rank and its effects on beef quality for animals raised under free conditions in mountain rangelands and relocated afterwards into a feedlot system. It is important to develop grouping management strategies and avoid regrouping during transportation.

Future studies should focus on elucidating the social stress in cattle when dominance hierarchies are being established and how they relate to beef quality. Further, to investigate how management can help avoid social stress and improve the quality of beef from animals being moved from rangeland to indoor conditions.

## Figures and Tables

**Table 1:** Social rank is defined by the Dominance Index (DI), and the description of the evaluated behaviors is shown below.

(A)		(B)						
Behavior	Description	Animal ID	Hierarchy 72 h		Ranking 6 Weeks		Hierarchy in Slaughterhouse	
			DI	Range	DI	Range	DI	Range
Head	When an animal is charging, hitting, or pushing another with its forehead, horns, or the base of its horns with a forceful movement and the recipient does not leave its current position.	1	0.83	D	0.77	D	1	D
		2	0.51	S	0.68	D	0.5	S
		3	0.68	D	0.66	D	0.33	S
		4	1	D	0.63	D	0.33	S
		5	0.44	S	0.25	S	0.36	S
Displace	After a headbutt, the recipient relinquishes its position by walking the distance of half an animal’s width or the distance of an animal’s width to one side.	6	0	S	0.45	S	0	S
		7	0.11	S	0.08	S	1	D
		8	0.35	S	0.49	S	0.8	D
		9	0.5	S	0.23	S	0.63	D
		10	0.55	S	0.29	S	1	D
Fight	Two animals vigorously push their heads, foreheads, horn bases, and/or horns against each other.	11	0.86	D	0.86	D	0.71	D
		12	0.14	S	0.52	S	0	S
		13	0.77	D	0.88	D	0.5	S
		14	0.63	D	0.45	S	0.5	S
		15	0.14	S	0.06	S	0.07	S
Continue	An animal makes another flee by following it quickly or running after it; this may be accompanied by sudden head movements.	16	0.3	S	0.76	D	0	S
		17	0.65	D	0.69	D	0.5	S
		18	0.86	D	0.69	D	0.43	S
		19	0.49	S	0.63	D	0.2	S
		20	0.04	S	0.27	S	0.76	D

Adapted from [19,20,21].

**Table 2:** Physicochemical characteristics of beef per animal.

ID	RBA	pH 45 min	pH 24 h	pH 0 d	SF 0 d	Final SF	WHC 0 d	Final WCH	Drip 0 d	L* 0 d	a* 0 d	b* 0 d	HUE 0 d	C* 0 d	Final L*	Final a*	Final b*	Final HUE	Final C*	∆E
1	10.4	6.57	5.53	5.44	3.15	1.56	62.7	72.7	1.16	46.8	16.4	6.89	22.8	17.8	47.7	15.8	6.88	23.5	17.3	0.97
2	10	6.45	5.2	5.34	3.97	2.75	59.6	71.4	1.38	43.5	21	12.8	31.5	24.6	38.3	16.5	5.74	19.2	17.5	9.88
3	9.6	6.75	5.58	5.21	3.06	2.71	62.4	69.3	1.23	45.8	19.5	11.9	31.5	22.9	39.6	14.9	6.2	22.6	16.1	9.63
4	9.1	6.42	6.19	5.46	3.26	1.85	62.5	63.5	1.11	41.1	17.4	8.14	25.1	19.2	42.9	16.1	4.78	16.6	16.8	4.01
5	9.75	6.76	5.64	5.21	3.04	3.15	60	75.2	1.38	44.9	18.2	12.5	34.6	22.1	40.8	14.9	5.24	19.4	15.7	9.02
6	7.25	6.62	5.29	5.5	2.44	2.18	60.1	73.6	1.31	41.6	21.8	11.9	28.7	24.9	44.9	19.1	7.34	21	20.5	6.26
7	9.1	6.36	4.83	5.3	3.22	2.35	64.3	66.7	1.78	45.5	21.4	13.9	32.9	25.5	44.3	16.3	6.57	22	17.5	9.01
8	10.9	6.41	5.56	5.51	2.93	1.99	58.4	69.8	1.55	45.8	19	11.3	30.8	22.1	44.9	17.1	5.56	18.1	17.9	6.14
9	7.4	6.33	5.42	5.28	3.09	3.47	60	69.5	1.47	44.7	19	12	32.2	22.5	44.7	15.4	6.27	22.2	16.6	6.77
10	8.55	6.53	5.93	5.3	4.37	4.03	63	65.8	1.68	43.5	20.3	12.4	31.5	23.8	44.7	17.5	7.09	22	18.9	6.15
11	10.7	6.25	5.2	5.41	2.84	2.39	54.8	70.2	1.51	43	15.7	9.96	32.4	18.6	45.9	14.3	7.51	27.8	16.1	4.01
12	9.1	6.18	5.59	5.39	5.71	3.92	58.5	67.5	1.45	48.3	17.5	9.39	28.2	19.9	43.5	17.3	8.07	25	19.1	5.01
13	8.3	6.69	5.73	5.33	3.51	2.99	64.3	72.2	1.09	42.1	18.9	10.7	29.5	21.7	43	16.6	6.65	21.9	17.8	4.72
14	10.9	6.44	5.48	5.48	3.07	2.35	62.8	68.3	1.43	42.7	19.5	10.3	27.8	22	43.4	16.1	8.06	26.6	18	4.07
15	11.2	6.02	5.6	5.28	2.19	1.8	61.7	67.2	1.23	44.6	22.7	13.4	30.6	26.4	46	17.8	8.87	26.5	19.9	6.87
16	7.95	6.18	4.93	5.36	2.7	2.18	61.6	72.9	1.09	43.6	17.1	10.3	31	20	45.1	15.9	6.84	23.3	17.3	3.99
17	9.55	6.41	5.46	5.49	2.71	2.23	59.1	71.3	1.79	44.3	20.7	12.3	30.8	24.1	44.6	17.9	6.89	21.1	19.2	6.1
18	9.45	6.65	5.28	5.46	2.73	2.22	63.6	66.8	1.27	42.4	16	9.02	29.4	18.4	42.3	17.9	7.03	21.4	19.2	2.73
19	10.8	6.41	5.42	5.37	3.88	2.73	67	70.6	1.76	43.3	15.8	9.98	32.3	18.7	39.5	15.6	4.84	17.2	16.4	6.4
20	11.1	6.46	4.96	5.36	4.33	3.14	63.1	65	1.29	45.5	22.6	13.6	31	26.4	45.8	16.7	7.52	24.3	18.3	8.47

RBA = rib eye area; pH45 = pH 45 min postmortem; pH24 = pH 24 h postmortem; 0 d = zero days on shelf; SF = shear force, WHC = water holding capacity (%); L* = luminosity; a* = red tendency; b* = yellow tendency; Hue = color tone; C* = chroma; Final = characteristics measured after 5 days on the shelf; ∆E, color difference between initial and final sampling.

## Data Availability

The dataset is available upon request from the authors.

## Abbreviations

The following abbreviations are used in this manuscript:

WHC	Water holding capacity
DI	Dominance Index
DM	Dominant
DS	Dominant to subordinate
SD	Subordinate to dominant
PCA	Principal component analysis
